# CD4^+^CD25^+^ T regulatory cells activated during feline immunodeficiency virus infection convert T helper cells into functional suppressors through a membrane-bound TGFβ / GARP-mediated mechanism

**DOI:** 10.1186/1743-422X-11-7

**Published:** 2014-01-18

**Authors:** Michelle M Miller, Christopher S Petty, Mary B Tompkins, Jonathan E Fogle

**Affiliations:** 1 Immunology Program, North Carolina State University, Raleigh NC, USA; 2 Department of Population Health and Pathobiology, North Carolina State University College of Veterinary Medicine, Raleigh NC 27607, USA; 3 Current address: Immunotherapy Technologies, LLC, 621 Hutton Street, Suite 105, Raleigh NC 27606, USA; 4 College of Veterinary Medicine, North Carolina State University, 1060 William Moore Drive, Raleigh, NC, 27607, USA

**Keywords:** FIV, HIV, AIDS, Lentivirus, Treg cells, mTGFβ, GARP

## Abstract

**Background:**

We and others have previously reported that cell membrane-bound TGFβ (mTGFβ) on activated T regulatory (Treg) cells mediates suppressor function. Current findings suggest that a novel protein known as Glycoprotein A Repetitions Predominant (GARP) anchors mTGFβ to the Treg cell surface and facilitates suppressor activity. Recently, we have described that GARP^+^TGFβ^+^ Treg cells expand during the course of FIV infection. Because Treg cells are anergic and generally exhibit poor proliferative ability, we asked how Treg homeostasis is maintained during the course of feline immunodeficiency virus (FIV) infection.

**Results:**

Here, we report that Treg cells from FIV^+^ cats express GARP and mTGFβ and convert T helper (Th) cells into phenotypic and functional Treg cells. Th to Treg conversion was abrogated by anti-TGFβ or anti-GARP treatment of Treg cells or by anti-TGFβRII treatment of Th cells, suggesting that Treg cell recruitment from the Th pool is mediated by TGFβ/TGFβRII signaling and that cell-surface GARP plays a major role in this process.

**Conclusions:**

These findings suggest Th to Treg conversion may initiate a cascade of events that contributes to the maintenance of virus reservoirs, progressive Th cell immunosuppression, and the development of immunodeficiency, all of which are central to the pathogenesis of AIDS lentivirus infections.

## Background

Thymus-derived T regulatory (Treg) cells are a distinct population of immunosuppressive CD4^+^ lymphocytes identified by constitutive expression of CD25 (IL2-R α-chain), GITR, CTLA-4 and the nuclear transcription factor, FoxP3 [1-3]. In addition to the well described Treg cells involved in self-tolerance, a population of pathogen-induced Treg cells has been described which express biologically active membrane TGFβ (mTGFβ) and play a major role in modulating immune responses to a variety of infectious agents by suppressing pathogen-induced CD4^+^ and CD8^+^ effector cells [4-7]. Expression of mTGFβ on activated Treg cells has recently been shown to be regulated by the glycoprotein A repetitions predominant (GARP) protein which is specifically expressed in the lymphoid compartment on regulatory cells and binds latent TGFβ to the Treg cell membrane [8-13]. Recent evidence has suggested that GARP functions in the conversion of latent TGFβ to biologically active TGFβ by enabling the cleavage of the latency associated peptide (LAP) of surface bound TGFβ by integrins (Wang 2012) [13]. However, it is not clear if this is the solitary mechanism for mTGFβ activation or if additional interactions occur during GARP:TGFβ association. We recently reported that TGFβ is anchored to the Treg cell surface by GARP and that GARP-anchored TGFβ is biologically active and capable of suppressing Th cell function [8]. Although there is considerable knowledge as to how mTGFβ^+^ Treg cells mediate suppression, there is less knowledge of the mechanism(s) that maintain their numbers and function in the peripheral immune compartment and how GARP may be involved. As Treg cells are anergic and exhibit limited ability to expand, there must be other factors maintaining their homeostasis [1,2,14,15]. Chen et al. [16] reported that CD4^+^CD25^-^ T cells stimulated via their TCR and treated with soluble TGFβ converted to a Treg cell phenotype, suggesting a mechanism for Th to Treg cell conversion. We previously reported that feline CD4^+^CD25^-^ Th cells could be converted to a Treg phenotype (CD25^+^mTGFβ^+^FoxP3^+^) by treatment with ConA and soluble TGFβ [17]. These converted cells displayed immunosuppressive function against ConA-stimulated CD4^+^CD25^-^ Th cells, suggesting that they possessed both the functional and phenotypic characteristics of activated Treg cells. To provide a mechanism for Th to Treg conversion, we demonstrated that ConA treatment of CD4^+^CD25^-^ Th cells up-regulates expression of TGFβRII on their surface, rendering them susceptible to Treg cell conversion by treatment with soluble TGFβ [17]. We also reported that anti-TGFβ receptor II (TGFβRII) treatment of ConA-stimulated Th cells abrogated the Th to Treg conversion, supporting a role for TGFβ/TGFβRII signaling in this conversion process [17]. Recent studies indicate that peripheral Treg cells, once activated, express both mTGFβ and GARP on their surface and that both molecules are instrumental in Treg cell suppressor function [11,12]. It is not known if this TGFβ/GARP complex plays a role in recruitment of Treg cells from the Th cell pool but evidence suggests that it may be integral to contact-dependent TGFβ signaling through TGFβRII [11,12].

The in vivo activation of Treg cells and subsequent suppression of CD4^+^ Th cells has been demonstrated in HIV and feline immunodeficiency virus (FIV) infection and likely represents an important component of lentiviral-induced immune suppression [4,5,18-20]. The exact mechanism underlying lentivirus-induced Treg cell activation is still unclear. However, we and others have previously demonstrated that CD4^+^CD25^+^ Treg cells are preferentially infected with FIV and activated during FIV infection [14,21-23]. Further, we have demonstrated that GARP bound mTGFβ is up-regulated on the activated Treg cell surface [8]. Many reports suggest that during the course of lentivirus infection the percentage of CD4^+^CD25^+^ cells among the CD4^+^ fraction remains stable, particularly in lymphoid organs, despite the overall decline in CD4^+^ cell numbers [4,20,24,25]. Therefore, a mechanism must exist by which CD4^+^CD25^+^ Treg cells are generated to maintain their homeostasis [1,2,24,26,27]. Several groups have demonstrated the ability of Treg cells to induce suppressor activity to Th cells, suggesting a mechanism for Treg cell recruitment and the maintenance of Treg cell homeostasis [28-30]. Based upon our work and work from others, we hypothesized that activated TGFβ^+^GARP^+^ Treg cells from FIV^+^ cats were capable of converting CD4^+^ Th cells to Treg cells, thereby maintaining Treg cells numbers and function during the course of FIV infection. Data presented herein support this hypothesis by first demonstrating that TGFβ and GARP are up-regulated on the surface of CD4^+^CD25^+^ Treg cells and TGFβRII is up-regulated on CD4^+^ Th cells from FIV-infected cats when compared to uninfected cats. Further, we show that co-culture of these two lymphocyte populations results in conversion of CD4^+^ Th cells into phenotypic and functionally immunosuppressive CD4^+^CD25^+^mTGFβ^+^GARP^+^FoxP3^+^ Treg cells. This Th to Treg conversion was blocked by either pretreatment of Treg cells with anti-TGFβ or anti-GARP antibodies or by pretreatment of target CD4^+^ Th cells with anti-TGFβRII antibodies. These results further support our hypothesis that one of the important mechanisms for maintaining peripheral Treg cell homeostasis is recruitment from the CD4^+^ Th cell pool in a TGFβ/GARP dependent manner. In the case of lentivirus infection, where both Treg and Th cells are chronically activated, maintenance of CD4^+^CD25^+^ Treg numbers and function may be favored at the expense of the Th cell pool. Therefore, chronic, progressive recruitment of immunosuppressive Treg cells from the Th cell pool could be an important factor contributing to both the maintenance of an activated Treg cell pool and the progressive Th cell immune deficiency characteristic of lentiviral infection.

## Results

### Membrane-bound TGFβ (mTGFβ) and GARP on the surface of CD4^+^CD25^+^ Treg cells and TGFβRII expression on CD4^+^CD25^-^ Th cells are increased during chronic FIV-infection

**Figure 1:**
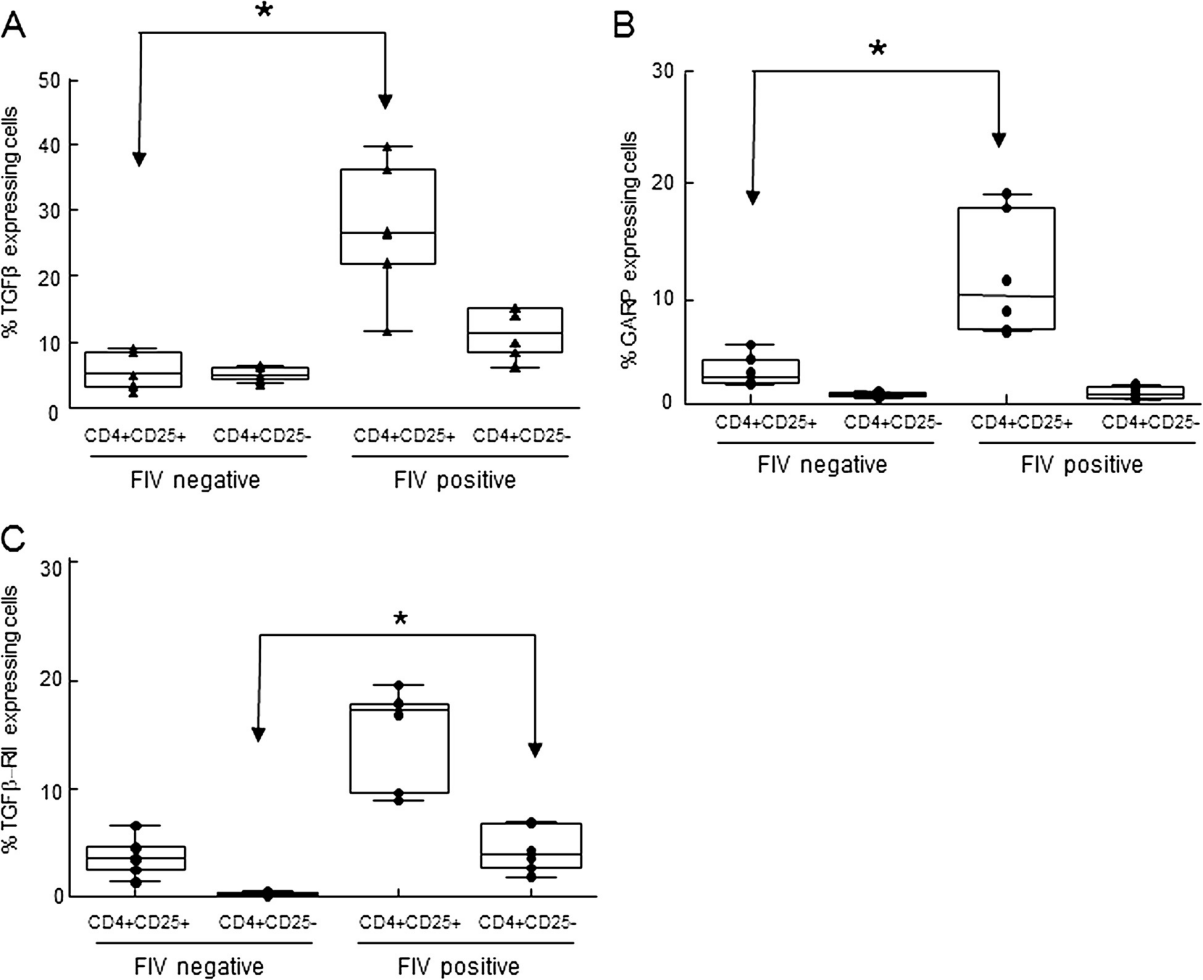
**PBMCs from FIV-infected cats display increased surface expression of TGFβ and GARP on CD4**^**+**^**CD25**^**+**^**lymphocytes and TGFβRII on CD4**^**+**^**CD25**^**-**^**lymphocytes.** PBMCs from FIV-infected or control cats were analyzed for surface expression of CD4, CD25, TGFβ, GARP and TGFβRII by flow cytometry. Cells were gated on CD4^+^CD25^+^ or CD4^+^CD25^-^ populations and analyzed for percent expression of **A**. TGFβ, **B**. GARP or **C**. TGFβRII. Box-whisker plots represent the 5^th^ and 95^th^ percentiles (whisker), 25^th^ and 75^th^ percentiles (box), and median of percent CD4^+^CD25^+^ and CD4^+^CD25^-^ expression from 6 FIV-positive and 6 FIV-negative cats. Symbols represent individual cats. CD4^+^CD25^+^ cells from FIV^+^ cats exhibit greater surface expression of TGFβ and GARP when compared to CD4^+^CD25^+^ cells from FIV^-^ cats. CD4^+^CD25^-^ cells from FIV^+^ cats exhibit greater surface expression of TGFβRII than CD4^+^CD25^-^ cells from FIV^-^ cats. (p <0.05, Mann–Whitney test for significance).

We have reported that ConA/TGFβ-stimulated CD4^+^CD25^-^ Th cells are converted to immunosuppressive CD25^+^mTGFβ^+^FoxP3^+^ Treg cells and that this conversion is abrogated by pre-treatment of ConA-stimulated Th target cells with anti-TGFβRII suggesting a TGFβ-TGFβRII mediated process [17]. Previously, Vahlenkamp, et al. [31] reported that CD4^+^CD25^+^ Treg cells from FIV^+^ cats are constitutively activated in vivo. We asked if these in vivo activated CD4^+^CD25^+^ Treg cells from FIV^+^ cats upregulate the activation markers GARP and mTGFβ. PBMC were isolated from 6 FIV negative cats and 6 chronically FIV-infected cats and analyzed for surface expression by flow cytometry. As shown in Figure 1, a significant increase in the percentage of CD4^+^CD25^+^ T cells expressing both GARP and mTGFβ was observed in FIV-infected cats when compared to FIV negative control cats (Figure 1A and B). Given that long-term FIV infection is associated with chronic T cell activation and with increased Treg cell suppressor function, we hypothesized that mTGFβ on activated Treg cells mediates suppressor function against Th cells expressing TGFβRII [31-33]. To address this question, we analyzed the expression of TGFβ-RII on the surface of CD4^+^CD25^-^ cells from FIV^+^ cats. As shown in Figure 1C, there is a significant increase in the percentage of CD4^+^CD25^-^ T cells from FIV^+^ cats expressing TGF-βRII when compared to control cats.

### CD4^+^CD25^+^ Treg cells from FIV^+^ cats convert CD4^+^CD25^-^ Th cells to a Treg phenotype

**Figure 2:**
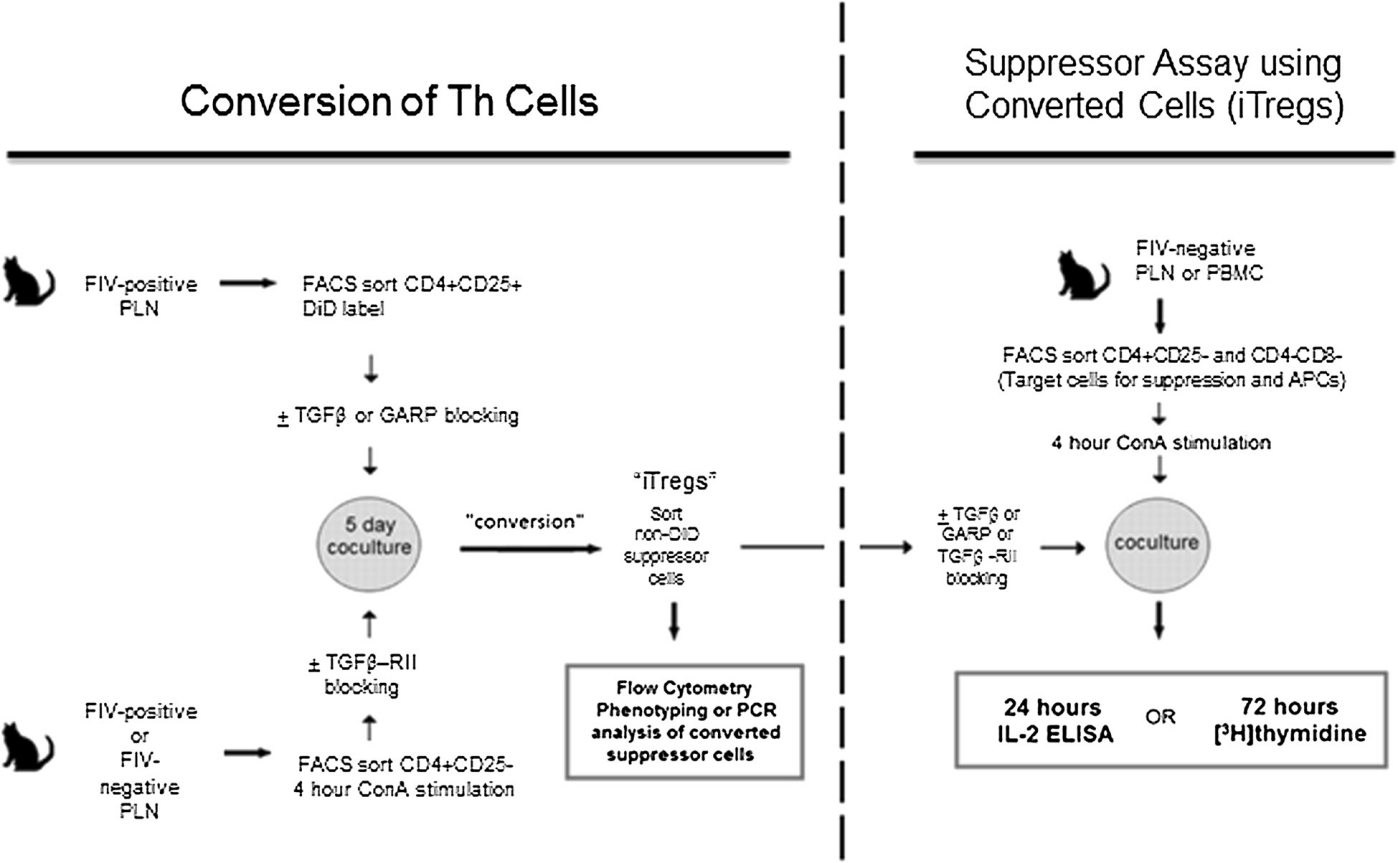
**Experimental design for CD4**^**+**^**CD25**^**+**^**Treg cell-mediated conversion of feline CD4**^**+**^**CD25**^**-**^**T cells to iTreg cells.** Single-cell suspensions were prepared from PLNs collected from FIV-infected or uninfected control cats. Cells were stained with anti-feline CD25 FITC and anti-feline CD4 PerCP labeled antibodies and CD4^+^CD25^+^ T cell populations purified by FACS. Converter CD4^+^CD25^+^ cells from control cats were stimulated with LPS/IL2 for 4 days, whereas CD4^+^CD25^+^ cells from FIV-infected cats are constitutively active and therefore were not stimulated prior to co-culture. PLN cells from FIV negative cats were FACs purified into CD4^+^CD25^-^ Th target cells or CD4^-^CD8^-^ APCs and stimulated for 4 hours with ConA. Converter cells were labeled with Vybrant DiD membrane dye and co-cultured with the purified CD4^+^CD25^-^ Th cells at a converter to target cell ratio of 1:2 in medium supplemented with 100 U/mL IL2. After 5 days, DiD membrane negative cells were FACS purified from the co-culture and analyzed for phenotype and function. In some experiments, anti-TGFβ, anti-GARP, or anti- TGFβRII antibodies were added prior to the conversion assay or prior to the functional assay.

Chen et al. [16] demonstrated that soluble TGFβ treatment coupled with TCR co-stimulation of Th cells induces a Treg phenotype and suppressor function. We also reported a similar in vitro Th to Treg conversion of feline ConA-stimulated CD4^+^CD25^-^ treated with soluble TGFβ [17]. There are a handful of reports describing the ability of Treg cells to induce suppressor activity in Th cells [28-30]. Increased expression of TGFβ on Treg cells and TGFβRII on Th cells could promote interaction between these two T cell subsets and facilitate the conversion of Th cells to Treg cells. To test this, freshly isolated, constitutively active CD4^+^CD25^+^ Treg cells from FIV^+^ cats were membrane-labeled with Vybrant DiD then co-cultured with ConA-activated, non-labeled CD4^+^CD25^-^ Th target cells as illustrated in Figure 2. After 5 days of co-culture, the DiD positive cells were depleted by FACS and the phenotype and suppressor function of the remaining cells, referred to herein as induced Treg (iTreg) cells, were analyzed. The positive control for conversion consisted of CD4^+^CD25^-^ cells from FIV negative cats cultured with ConA and soluble TGFβ which induces FoxP3 expression and the acquisition of suppressor function in feline Th cells as described previously [17]. Negative controls consisted of CD4^+^CD25^-^ cells cultured in medium alone or in medium supplemented with either 5 ug/mL ConA or 100 U/mL IL2.

**Figure 3:**
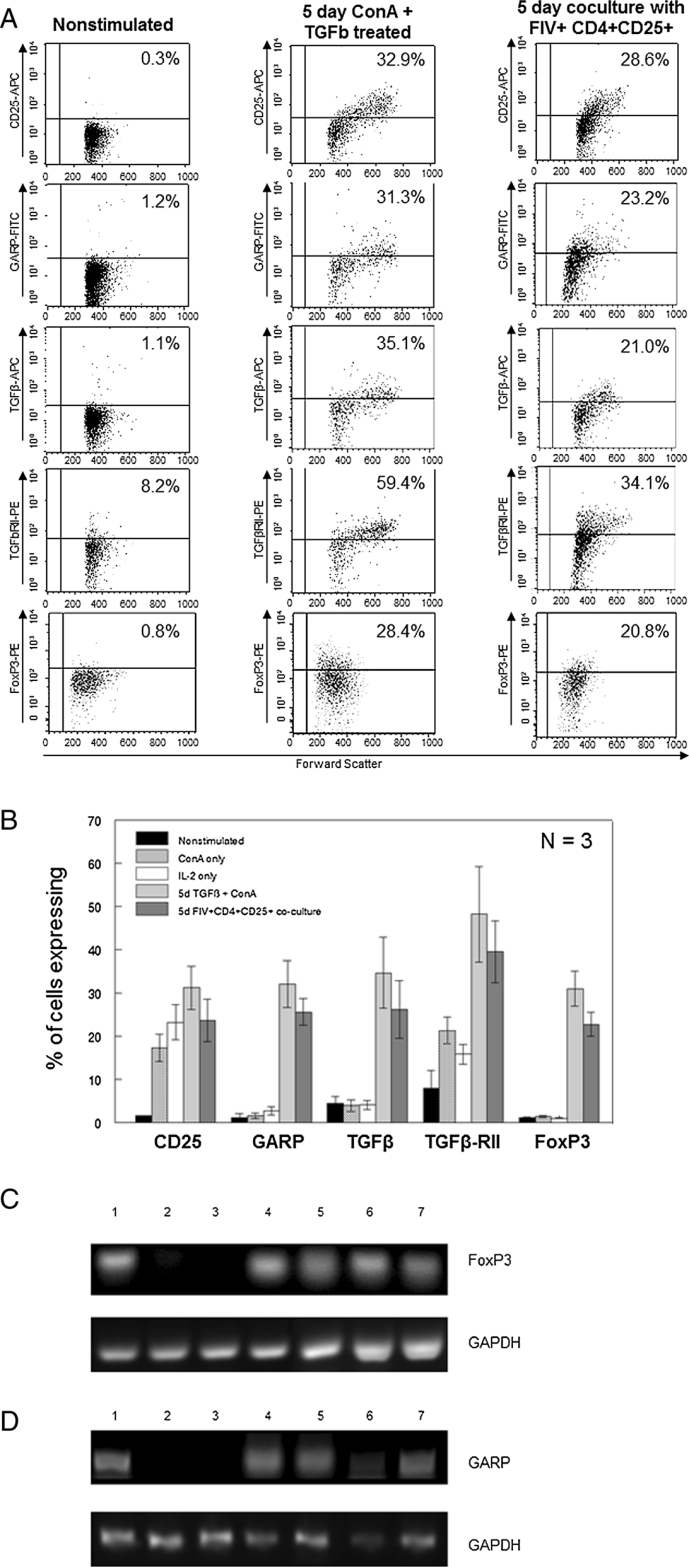
**CD4**^**+**^**CD25**^**+**^**Treg cells from FIV-infected cats are capable of converting CD4**^**+**^**CD25**^**-**^**Th cells from FIV-negative cats into phenotypic iTreg cells. A**. Untreated CD4^+^CD25^-^ T cells, soluble TGFβ and ConA treated CD4^+^CD25^-^ cells, or CD4^+^CD25^-^ cells from control cats co-cultured with Treg cells from FIV^+^ cats were analyzed by flow cytometry for the Treg surface phenotypic markers CD25, GARP, TGFβ and TGFβRII after the 5 days in culture. Representative dot plots are shown. The percent of cells expressing each marker is at the top right of each dot plot. **B**. CD4^+^CD25^-^ cells treated as described for **(A)**, as well as CD4^+^CD25^-^ cells treated with IL2 alone or ConA alone were analyzed for expression of CD25, GARP, TGFβ, TGFβRII and FoxP3. Bars represent the mean ± SEM percent of cells expressing each marker from three independent experiments from separate cats. **C** and **D**. Target CD4^+^CD25^-^ cells from two FIV^+^ cats were cultured as described above then analyzed for FoxP3 **(C)** or GARP **(D)** mRNA expression by PCR. Lanes 1: freshly isolated CD4^+^CD25^+^ Treg cells from Cat A. Lanes 2 and 3: CD4^+^CD25^-^ cells from Cat A (2) or Cat B (3) cultured for 5 days with IL2 alone. Lanes 4 and 5: CD4^+^CD25^-^ cells from Cat A (4) and Cat B (5) cultured for 5 days with soluble TGFβ (10 ng/mL) and ConA (5 ug/mL). Lanes 6 and 7: ConA-activated CD4^+^CD25^-^ cells from Cat A (6) and Cat B (7) co-cultured for 5 days with autologous CD4^+^CD25^+^ then purified by FACS. The target cells cultured with sTGFβ/ConA or with activated Treg cells express FoxP3 and GARP mRNA.

Following culture of CD4^+^CD25^+^ PLN cells from FIV-infected cats with CD4^+^CD25^-^ cells from FIV negative cats, the latter population (iTreg cells) was analyzed by flow cytometry for the expression of CD25, GARP, TGFβ, TGFβRII and FoxP3. Figure 3A shows representative dot plots from one conversion assay and Figure 3B shows the averages of 3 experiments. Non-stimulated CD4^+^CD25^-^ Th cells do not express the Treg-associated surface proteins CD25, GARP, or TGFβ and less than 10% of these cells express TGFβRII, consistent with a resting Th population. Treatment of Th cells with ConA or IL2 alone resulted in an increased percentage of cells expressing CD25 and TGFβRII, suggesting Th cell activation, but there was no increase in cells expressing the Treg cell markers GARP, mTGFβ or FoxP3 (Figure 3A-B). Only culturing in the presence of ConA and TGFβ, as we have previously demonstrated, or culturing with activated Treg cells from FIV^+^ cats resulted in the expression of GARP, mTGFβ, and FoxP3, as well as CD25 and TGFβRII, on approximately one third of the Th cells. As the stable expression of forkhead transcription factor FoxP3 is the defining marker of CD4^+^CD25^+^ Treg cells and is required for both homeostasis and suppressor function, we also analyzed FoxP3 mRNA expression in Th cells converted to iTreg cells [3]. Similar to the flow cytometry analysis, only Th cells cultured in the presence of ConA and TGFβ or with activated Treg cells from FIV^+^ cats led to the expression of FoxP3 or GARP mRNA (Figure 3C and D). This phenotype (CD25^+^GARP^+^mTGFβ^+^FoxP3^+^) is consistent with Treg cells and suggests that activated Treg cells are capable of converting Th cells to a Treg phenotype.

### CD4^+^CD25^+^ T cells from FIV-infected cats convert CD4^+^CD25^-^ Th cells into iTreg cells capable of suppressing Th cell IL2 production and proliferation

**Figure 4:**
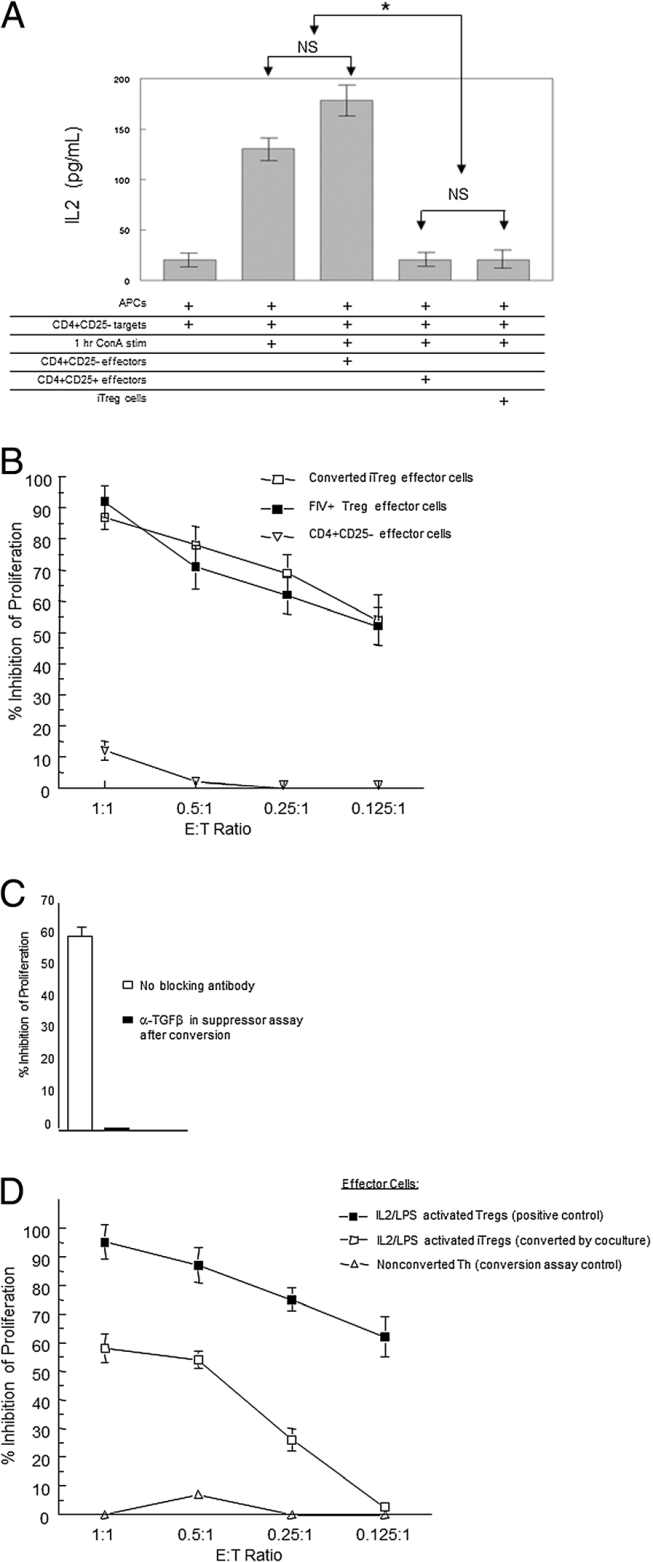
**Activated feline CD4**^**+**^**CD25**^**+**^**T cells from FIV-infected cats convert CD4**^**+**^**CD25**^**-**^**Th cells into functional iTreg cells.** CD4^+^CD25^-^ cells were converted by co-culture with CD4^+^CD25^+^ T cells (outlined in Figure 2) and effector function analyzed. For suppression assays, CD4^+^CD25^-^ (Th) and CD4^-^CD8^-^ (APCs) from control cats were combined at a 1:1 ratio for use as targets and, where indicated, stimulated for 1 hour with ConA (5 ug/uL). **A**. Nonstimulated CD4^+^CD25^-^ (negative control), LPS/IL2 activated CD4^+^CD25^+^ (positive control), or iTregs from conversion co-cultures with FIV^+^ Tregs were used as effectors. After 24 hours, IL2 was analyzed in the supernatent by ELISA in triplicate. Data shown is representative of two experiments. Error bars represent the SEM (*p < .05 Mann–Whitney). **B**. CD4^+^CD25^-^ target cells were co-cultured with iTregs (open square), or freshly isolated CD4^+^CD25^+^ cells from an FIV^+^ cat (closed square), or CD4^+^CD25^-^ cells (inverted triangle) at various E:T ratios for 72 hrs and pulsed with ^3^H-thymidine. Symbols are the mean ± SEM percent inhibition of proliferation of targets from three experiments. **C**. iTregs either untreated or pre-incubated with anti-TGFβ were used as effectors in a proliferation assay as described in B. Bars represent the mean ± SEM percent inhibition of proliferation of Th targets for three experiments. **D**. CD4^+^CD25^-^ targets for the iTreg conversion assay were first cultured for 5 days with DiD-labeled CD4^+^CD25^+^ Tregs isolated from an FIV negative cat and activated by 5 day IL2/LPS treatment. Following co-culture, converted cells were isolated by FACS and used as effectors in a standard 72 hour ^3^H-thymidine proliferation assay with CD4^+^CD25^-^ targets at various E:T ratios. Effector cell control for the assay was 5 day IL2/LPS activated CD4^+^CD25^+^ Tregs (closed square, positive control). Bars represent the mean ± SEM percent inhibition of proliferation of targets and represent three separate experiments.

Figure 3 demonstrates that Th cells can be induced to express surface and intracellular markers consistent with a Treg cell phenotype. Therefore, we asked if these converted Th cells were truly functional suppressor cells. ConA-stimulated CD4^+^CD25^-^ target cells were co-cultured with iTreg effector cells at a 1:2 effector to target ratio (iTreg to CD4^+^ Th) for 24 hours, then culture supernatant was collected and analyzed for IL2 by ELISA. As shown in Figure 4A, addition of iTreg cells to CD4^+^ Th cultures from FIV^+^ cats (fifth column) resulted in a significant reduction of IL2 in the culture supernatant which was similar to that of Treg cells directly isolated from FIV^+^ cats (fourth column). As shown in Figure 4A, addition of unconverted CD4^+^CD25^-^ cells to the CD4^+^ Th culture resulted in a slight enhancement of IL2 in the culture supernatant. iTreg suppressor function was also assayed by a standard proliferation assay of ConA-activated CD4^+^CD25^-^ target Th cells. The suppressor function of the converted iTreg cells was similar to suppression by isolated CD4^+^CD25^+^ Treg cells from FIV^+^ cats (Figure 4B, open squares, closed squares). In addition, pre-treatment of iTreg cells with anti-TGFβ effectively eliminated the ability of iTreg cells to inhibit the proliferation of activated CD4^+^CD25^-^ T cells (Figure 4C). These results demonstrate that in addition to displaying the phenotypic characteristics of a regulatory T cell population, the iTreg cells produced from co-culture of Th cells with Treg cells from FIV^+^ cats have functional characteristics of Treg cells.

We have demonstrated previously that IL2 and LPS treatment is required to activate CD4^+^CD25^+^ Treg cells from FIV^-^ cats [8,31]. To confirm that conversion of CD4^+^CD25^-^ cells into iTreg cells was due to activated Treg cells and not an artifact of co-culture conditions (IL2, LPS, ConA) or an indirect effect of FIV infection, Th cells were co-cultured with DiD stained CD4^+^CD25^+^ cells from FIV negative control cats treated prior to co-culture with IL2/LPS. After the 5 day co-culture, the DiD “converter” population was depleted and the remaining cells were assayed for effector function using the [H^3^]-Thymidine assay for proliferation of activated Th target cells. Like constitutively activated Treg cells from FIV^+^ cats, in vitro activated CD4^+^CD25^+^ cells from FIV^-^ cats converted Th cells into functional iTreg cells as measured by inhibition of proliferation of ConA stimulated Th target cells (Figure 4D, open squares) but were not quite as efficient at suppression as directly isolated CD4^+^CD25^+^ Treg cells (Figure 4D, closed squares, positive control). Conversion did not appear to be an artifact of co-culture as CD4^+^CD25^+^ cells treated with IL2 alone were not capable of inducing iTreg function in Th target cells (iTreg conversion control, not shown). Likewise, CD4^+^CD25^-^ cells treated with IL2/LPS (triangles, Th suppression assay control), or IL2 alone (not shown) did not exhibit inhibition of Th target cell proliferation.

### Blockade of TGFβ/TGFRII Interaction Abrogates CD4^+^CD25^+^ Treg-Induced Conversion of CD4^+^CD25^-^ Th cells to iTreg cells

**Figure 5:**
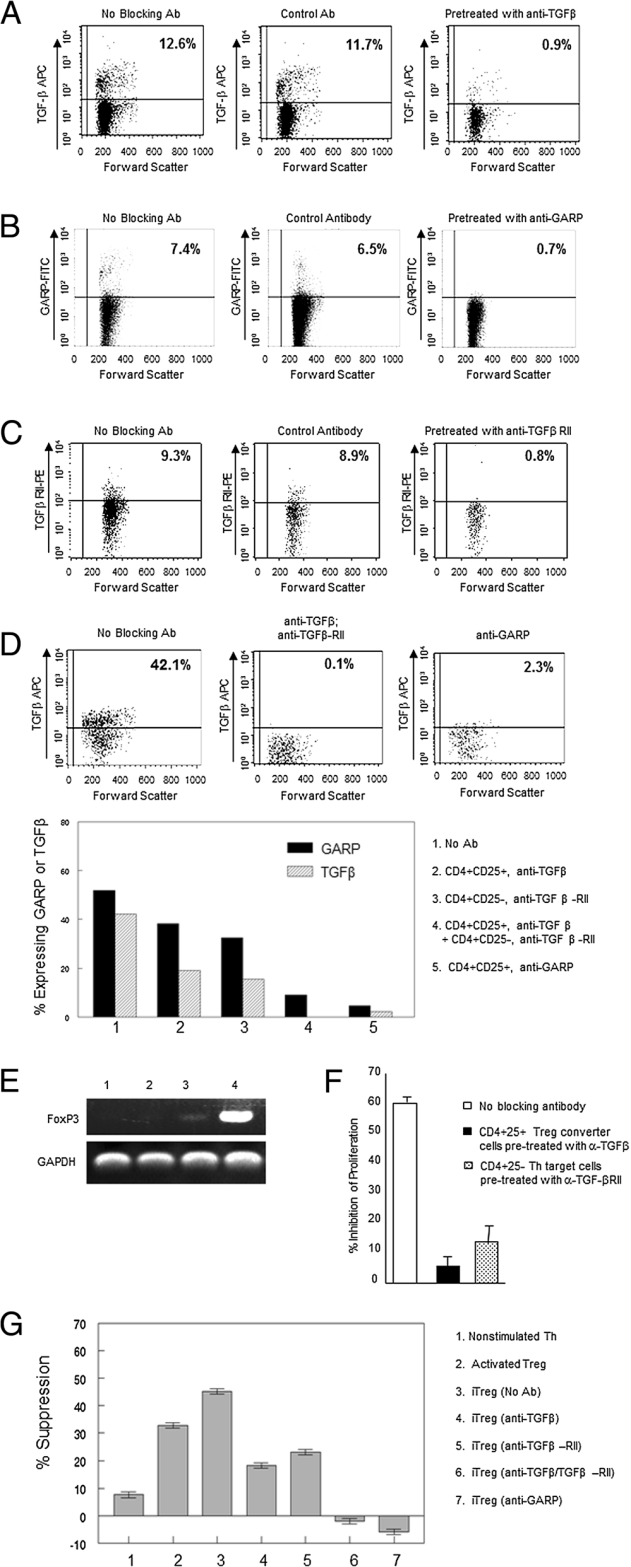
**Feline CD4**^**+**^**CD25**^**+**^**T cell-mediated iTreg conversion occurs by a TGFβ/TGFβRII dependent mechanism. A-C**. To confirm blocking of TGFβ **(A)** or GARP **(B)** on CD4^+^CD25^+^ Treg cells and TGFβRII **(C)** on CD4^+^CD25^-^ Th cells, cells were pretreated with unconjugated specific or isotype control antibody, then analyzed by flow cytometry using conjugated antibody to confirm blocking of TGFβ and GARP on CD4^+^CD25^+^ and TGFβRII on CD4^+^CD25^-^ cells. Data represent three experiments. **D-G**. ConA-activated Th targets were pre-treated with anti-TGFβRII antibodies and/or Tregs were pre-treated with anti-TGFβ or anti-GARP antibodies for 30 min, washed, co-cultured for 5 days, then target cells were analyzed for induction of Treg phenotype and function. **D**. Dot plots demonstrating surface TGFβ expression following conversion of Th cells to iTregs and blocking TGFβ/TGFβRII or GARP prior to co-culture (upper panels). Surface expression of both (1) GARP and TGFβ were decreased by blocking (2) TGFβ, (3) TGFβRII, (4) TGFβ/TGFβRII, or (5) GARP (lower panel). Data represents one blocking study. **E**. Treatment of CD4^+^CD25^+^ converter cells with anti-TGFβ (lane 1) or anti-GARP (lane 2) or of CD4^+^CD25^-^ target cells with anti-TGFβRII (lane 3) prior to co-culture prevented induction of FoxP3 in Th targets. Lane 4: Th targets with no antibody blockade. **F**, **G**. Suppressor function of iTregs was evaluated by ^3^H-thymidine proliferation **(F)** or by IL2 suppression assay **(G)**. Suppressor function of iTregs was reduced by anti-TGFβ treatment of converter Treg cells (**F** black bar, **G** lane 4) or by anti-TGFβRII treatment of target Th cells (**F** pattern bar, **G** lane 5) prior to conversion. Conversion was completely abrogated when TGFβ and TGFβRII were blocked simultaneously or when GARP was blocked during conversion (**G** lanes 6 and 7). Data in **F** represents mean + SEM of three experiments; **G** mean ± SEM of experimental triplicates.

To confirm that CD4^+^CD25^+^ Treg cells mediate conversion of CD4^+^CD25^-^ Th to iTreg cells by a TGFβ/TGFβRII signaling pathway, DiD labeled CD4^+^CD25^+^ Treg cells from FIV-infected cats were pre-treated with anti-TGFβ or anti-GARP, or ConA activated CD4^+^CD25^-^ Th target cells were pre-treated with anti-TGF-RII antibodies prior to co-culture. Flow cytometric analysis demonstrated that pretreatment of CD4^+^CD25^+^ cells with anti-TGFβ (Figure 5A) or anti-GARP (Figure 5B) or pre-treatment of CD4^+^CD25^-^ cells with anti-TGFβRII (Figure 5C) for 15 minutes prior to flow cytometry analysis prevented the binding of fluorochrome-labeled detection antibodies, demonstrating efficient blocking of these surface proteins. After blocking TGFβ or GARP on DiD-labeled CD4^+^CD25^+^ cells or TGFβRII on CD4^+^CD25^-^ cells, cells were co-cultured then DiD^+^ cells depleted, as described for in vitro conversion previously. The remaining cells were analyzed for phenotypic and functional characteristics of iTreg cells. Flow cytometry analysis revealed that anti-TGFβ or anti-GARP pre-treatment of CD4^+^CD25^+^ T cells, and/or anti-TGFβRII pre-treatment of CD4^+^CD25^-^ cells prior to co-culture significantly reduced the expression of TGFβ and GARP on the surface of the CD4^+^ Th cells after co-culture, suggesting failure to convert Th cells to iTreg cells (Figure 5D). In addition, anti-TGFβ or anti-GARP treatment of Treg cells, or anti-TGFβRII treatment of Th cells prevented the expression of FoxP3 mRNA in the Th target population (Figure 5E), suggesting that mTGFβ and GARP on Treg cells and TGFβRII on Th cells are crucial for the Treg-mediated conversion of CD4^+^CD25^-^ Th cells to an iTreg phenotype.

Blocking mTGFβ on Treg converter cells (Figure 5D, bar 4) or blocking TGFβRII on Th target cells (Figure 5D, bar 5) before co-culture abrogated the induction of suppressor positive iTreg cells as measured by proliferation (Figure 5 F) and IL2 production (Figure 5G, bars 4 and 5) by CD4^+^ Th cells. Blocking of TGFβ on converter cells in conjunction with anti-TGFβRII on activated Th target cells completely inhibited the induction of functional iTreg cells (Figure 5G, bar 6). Blocking of GARP alone on converter cells prior to co-culture also completely eliminated the induction of functional iTreg cells (Figure 5G, bar 7). Thus, the conversion of Th cells to phenotypic and functional iTreg cells is dependent upon GARP-bound TGFβ and TGFβRII signaling.

## Discussion

It is well established that pathogen-induced Treg cells play a central role in maintaining the balance between immunity and immunopathology to infectious agents in the peripheral immune compartment [1,4,5]. However, mechanisms regulating their homeostasis and suppressor function remain unclear. This is particularly important in the case of AIDS lentivirus infection, as Treg cells are chronically activated and in many cases their numbers seem to be maintained, even in the face of declining total CD4^+^ T cell numbers [4,20,24,25]. These observations suggest there is a mechanism(s) for maintaining Treg cell peripheral homeostasis and suppressor function, as they do not produce IL2, are anergic, and expand only under specific conditions [15,31]. While a number of mechanisms have been proposed to explain Treg cell homeostasis, evidence supports recruitment of de novo suppressor cells from the Th cell pool [28-30]. As it has been established that Treg cells are chronically activated and constitutively immunosuppressive throughout the course of FIV and HIV infections, we examined the possibility that these activated Treg cells in FIV-infected cats are capable of converting CD4^+^ Th cells into a Treg phenotype (iTreg cells), thereby maintaining population numbers [34,35]. This is hypothesis supported by the observations that Treg cell homeostasis can be maintained in the periphery during responses to pathogens such as hepatitis viruses, add a comma after viruses SIV, HIV, and FIV and molecules such as LPS and IL2 have been shown to induce Treg proliferation [31,35]. Chen et al. [16] reported that CD4^+^CD25^-^ Th cells could be converted into FoxP3^+^ immunosuppressive Treg cells in vitro by stimulation with soluble TGFβ in combination with TCR engagement, suggesting a role for TGFβ. These findings are supported by other reports that soluble TGFβ of activated TGFβRII^+^ Th target cells in mice induces suppressor activity [36,37]. Our laboratory has also demonstrated that stimulation of CD4^+^CD25^-^ Th cells with a combination of ConA and TGFβ converted these cells into CD25^+^mTGFβ^+^FoxP3^+^ functional suppressor cells [17]. Taken together, these studies indicate that Treg peripheral homeostasis may be maintained by recruitment from the CD4^+^CD25^-^ Th cell population in a TGFβ-dependent process.

In support of Th to Treg conversion in vivo, we demonstrated that, in contrast to Treg cells from uninfected cats, a significant number of Treg cells in FIV^+^ cats are mTGFβ positive and that a fraction of Th cells express TGFβRII. To examine the concept that in vivo Th to Treg conversion could occur in the peripheral lymphoid tissue of FIV^+^ cats by engagement of TGFβ on Treg cells with TGFβRII on CD4^+^ Th target cells, an ex vivo model for conversion was designed. ConA-activated TGFβRII^+^CD4^+^CD25^-^ Th cells from control cats co-cultured with CD4^+^CD25^+^ Treg cells from FIV^+^ cats displayed the Treg-cell markers CD25 and mTGFβ and expressed immunosuppressor function (iTreg cells), consistent with a Treg cell phenotype. As others have reported that TGFβ regulates the expression of FoxP3 in Treg cells and as FoxP3 is required for Treg homeostasis and suppressor function we analyzed iTreg cells for FoxP3 expression [38-40]. The iTreg cells induced by co-culture with autologous Treg cells markedly up-regulated FoxP3 mRNA. We also examined surface GARP expression on the iTreg cells, as GARP has been reported to be unique to Treg cells and important for mTGFβ function by anchoring TGFβ to the cell surface [8-13]. We also previously reported that GARP is expressed in association with mTGFβ on activated Treg cells from FIV^+^ cats [8]. The results reported here demonstrate that constitutively activated Treg cells from FIV^+^ cats induced GARP mRNA and protein expression in Th cell targets following co-culture confirming that iTreg cells have an activated Treg phenotype. Although the observed Treg cell virus load during chronic infection is typically low, Treg cells are highly susceptible to FIV infection in vitro and a percentage of the cells isolated from FIV-infected cats for the conversion assays may have carried FIV, raising the question of whether Th conversion in these cocultures was a result of de novo infection in the target cells. However, given the slow rate of growth inherent to lentiviruses only a very small fraction of T cells would become newly infected during a 5 day coculture. Therefore, while this may still contribute to some of the activation and conversion observed, it would not account for the nearly 50% conversion rate demonstrated by the phenotypic analysis reported in Figure 3.

As shown in Figure 4, iTreg cells are functionally activated, as they are capable of suppressing IL2 production and proliferation by activated CD4^+^CD25^-^ Th cells in vitro at a level comparable to that of constitutively activated Treg cells from FIV^+^ cats. Additionally, these iTreg cells suppress activated Th cell proliferation by the same TGFβ-mediated mechanism as non-induced Treg cells. In this regard, we and others have demonstrated that blocking of TGFβ on activated Treg cells or blocking of TGFβ-RII on Th target cells abrogates Treg suppressor function, suggesting that Treg suppression is mediated by a TFGβ/TGFβRII signaling pathway [8,17,36]. In this study, addition of anti-TGFβ or anti- TGFβRII antibody to the suppressor assay for iTreg cells inhibited the suppressor function of these cells, supporting the role of TGFβ/TGFβRII in mediating suppression. The size of the Treg cell compartment, FoxP3 expression, and suppressor function in CD4^+^ T cells have been shown to be dependent on signals induced by TGFβ in the periphery [39]. In concert with these findings, the data presented in this study demonstrate that engagement of mTGFβ on Treg with TGFβRII on Th cells converts the latter into iTreg cells in FIV^+^ cats. Importantly, conversion of Th cells was also observed following coculture with activated Treg cells from noninfected cats as shown in Figure 4D. This experiment demonstrates that the conversion of iTreg cells in these coculture conditions is not an artifact of infection status or chronic FIV antigen presentation and suggests that Treg cells are converting Th cells by a more direct interaction such as mTGFβ signaling. The role of the TGFβ/TGFβRII signaling pathway in the conversion of Th to iTreg cells was confirmed by the observation that pretreatment of activated Treg cells with antibodies to TGFβ or GARP or pretreatment of activated Th cells with anti-TGFβRII blocked the conversion of Th cells to an iTreg phenotype. Dieckmann et al. [28] reported that activated CD4^+^CD25^+^ Treg cells are capable of suppressing both CD4^+^ and CD8^+^ T cells but induce a Treg phenotype only in activated Th cells and not in CD8^+^ T cells. Similar to those findings, we also have demonstrated that constitutively activated Treg cells from FIV^+^ cats suppress both CD4^+^ Th and CD8^+^ T cell responses, but do not induce CD8^+^ T cell suppressor function [18,20,28]. The results of the studies reported here clearly demonstrate that activated Tregs induce suppressor function in CD4^+^ Th cells. To our knowledge, this is the first report describing Th to iTreg cell conversion by lentivirus-activated Treg cells.

## Conclusions

Collectively, these data demonstrate that peripheral Treg homeostasis may be regulated by recruitment from the Th cell pool by mTGFβ/TGFβ-RII signaling. We believe lentivirus infection may promote this process and disrupt the normal balance between Th and Treg cells by chronic activation of both Treg and Th cells, tipping the equilibrium in favor of iTreg cells at the expense of the Th cell pool. Thus Th to iTreg cell conversion that is mediated by activated CD4^+^CD25^+^ mTGFβ^+^FoxP3^+^ Treg cells may limit expansion and effector function of anti-viral CD4^+^ T cells by converting them to CD25^+^mTGFβ^+^FoxP3^+^ iTreg cells, contributing to the Treg cell pool during the progression of AIDS lentivirus infections. This is an important concept, as the relative balance between CD4^+^ Th cell immune responses and CD4^+^CD25^+^ Treg immune suppression during the acute stage of virus infection may help to determine the ultimate virus set point and the long-term ability to control viremia, and in effect predict disease progression. A more in depth understanding of conversion events and the emergence of iTreg populations during acute infection are instrumental in understanding this process. The data presented in this manuscript clearly demonstrates that FIV-AIDS lentivirus infection is capable of activating CD4^+^CD25^+^ Treg cells expressing mTGFβ that are, in turn, capable of interacting with TGFβRII^+^CD4^+^ Th cells and converting the Th cells into iTreg cells in vitro. These studies help explain both the maintenance of Treg numbers and function, and provide a possible mechanism for lentivirus-induced CD4^+^ T cell immune dysfunction. We are currently examining how this may occur in vivo during the course of FIV infection. While our data clearly demonstrate that GARP is involved in this conversion, the exact role of membrane associated GARP in functional activation of mTGFβ on Treg cells and iTreg cells remains to be determined. A recent report has suggested that GARP enables the activation of mTGFβ by providing a scaffold on the Treg cell surface, positioning the latent TGF molecule in such a way as to facilitate its interaction with integrins which cleave the latency associated peptide (LAP), producing biologically active mTGFβ (Wang 2012). The possibility remains that additional interactions, either at the Treg cell surface or during intracellular protein transport, are critical for this activation process. The full characterization and mapping of the mTGFβ activation process would be invaluable for designing future Treg studies and should be taken under careful consideration.

## Methods

### Cats and FIV infection

Specific pathogen-free cats were obtained from Liberty Labs (Liberty Corners, NJ) or Cedar River Laboratory (Mason City, IA) and housed at the Laboratory Animal Resource Facility at the College of Veterinary Medicine, North Carolina State University. Cats were inoculated with the NCSU_1_ isolate of FIV, a pathogenic clade A virus, as described by Bucci et al. [41]. FIV-infection was confirmed by immunoblot analysis and provirus detection by PCR using primers specific for the FIV-p24 GAG sequence. At the time samples were taken, cats had been infected with FIV for at least 5 years and were clinically asymptomatic. Non-infected control cats ranged in age from 3 to 6 years and were housed separately from FIV-infected cats. Protocols were approved by the North Carolina State University Institutional Animal Care and Use Committee.

### Sample collection and preparation

Whole blood (28 ml/cat) was collected by jugular venipuncture into EDTA Vacutainer tubes (Becton-Dickinson, Franklin Lakes, NJ). PBMC were isolated by Percoll density gradient centrifugation (Sigma-Aldrich, St. Louis, MO) as previously described [42] or by Ficoll-Histopaque-1077 density gradient centrifugation (Sigma-Aldrich, St-Louis, MO) following the manufacturer’s guidelines. Single-cell suspensions were prepared from popliteal or submandibular peripheral lymph nodes (PLN) obtained through surgical biopsies by gently and repeatedly injecting sterile PBS into the tissue using an 18G needle until the cells were released from the tissue. Cell counts and viability were determined by trypan blue dye exclusion and viability was always >90%.

### Reagents and antibodies

Recombinant human IL2 was obtained through the AIDS Research and Reference Reagent Program, Division of AIDS, NIAID, NIH from Dr. Maurice Gately, Hoffmann - La Roche Inc. LPS and Concanavalin A (ConA) were purchased from Sigma-Aldrich (St. Louis, MO). Anti-mouse IgG coated magnetic Dynabeads® M-450 were purchased from Dynal (Great Neck, NY). Streptavidin–PerCP was purchased from BD Biosciences PharMingen (San Diego, CA). Anti-TGFβ1 (MAB240) was purchased from R&D Systems (Minneapolis, MN) and conjugated to allophycocyanin (APC) or left unconjugated for blocking studies; PE-conjugated anti-TGFβ-RII (FAB241P), neutralizing anti-TGFβRII (AF-241-NA), and recombinant human TGFβ1 (240-B) were also purchased from R&D Systems. Mouse anti-feline CD25 (mAb 9 F23) was kindly provided by K. Ohno (University of Tokyo, Tokyo, Japan). Anti-CD21 was purchased from Serotec (Raleigh, NC). Mouse anti-feline CD4 (mAb 30A) and CD8 (mAb 3.357) were developed in our laboratory [43]. PE conjugated rat anti-mouse FoxP3 (FJK-16 s) was purchased from eBioscience (San Diego, CA). FITC-conjugated anti-GARP IgG2b monoclonal antibody (LRRC32, Plato-1) was purchased from Enzo life Sciences (Ann Arbor, MI).

### Flow cytometric analysis

For comparison of TGFβ and TGFβRII in chronic FIV-infected or uninfected cats, at least 5 × 10^5^ PBMC were stained for surface expression using specific antibodies and three-color flow cytometry was performed on a FACSCaliber flow cytometer (BD Biosciences, Mountain View, CA). Lymphocytes were gated based on forward vs. side scatter, and 20,000 gated events were acquired and stored list-mode fashion for analysis using CellQuest software. For phenotyping studies, sorted and/or cultured cells were stained for surface expression of CD4, CD25, TGFβ, GARP and TGFβRII. For intracellular staining of FoxP3, cells were first stained for CD4 and CD25 expression, washed in PBS, incubated with 4% PFA for 10 minutes, and washed twice more. Cells were then incubated in 0.1% Triton x-100 for 30 minutes, washed with PBS + 4% FBS, resuspended in 100 uL of PBS and incubated with FoxP3-specific antibody at room temperature for 20 minutes. Cells were washed in PBS + 4% FBS and analyzed on the FACSCalibur flow cytometer, lymphocytes were gates on forward vs. side scatter and 20,000 gated events were acquired and stored list-mode fashion for analysis using CellQuest software. All gating was determined by isotype controls.

### Purification of lymphocyte populations

CD4^+^CD25^+^ and CD4^+^CD25^-^ cell populations were purified as previously described [17,20]. Briefly, for FACS purification, PBMCs or LN cells were stained with anti-CD4, anti-CD25 and anti-CD8. CD4^+^CD25^+^, CD4^+^CD25^-^ and CD4^-^CD8^-^ (used as antigen presenting cells [APCs]) cell subsets were purified using a high-speed, high-purity fluorescence activated cell sorter (MoFlo, DakoCytomation). The purity of FACS sorted cell populations was always > 95%. CD4^+^CD25^-^ T cells for use as target cells in proliferation assays were enriched using biomagnetic bead separation using goat anti-mouse IgG-coated beads as described by Bucci et al. [41]. Briefly, CD21^+^ B cells, CD8^+^, and CD25^+^ T cells were depleted in successive steps using magnetic beads coated with anti-CD21, anti-CD8 and anti-CD25 antibody respectively. Purity of the magnetic bead enriched CD4^+^CD25^-^ T cells was > 90%, as verified by flow cytometric analysis.

### Treg cell-induced conversion assay

Converter cells consisted of purified CD4^+^CD25^+^ T cells from either control cat or FIV-infected cat PLN. In the case of control cats, the converter cells were activated by culturing (4 × 10^6^ per well, 24-well plate) for 4 days in the presence of LPS (10 μg/mL) plus IL2 (100 U/mL) then washed twice and labeled with Vybrant DiD (Molecular Probes) fluorescent dye. In the case of FIV-infected cats, as the CD4^+^CD25^+^ T cells are constitutively activated, the converter cells were freshly isolated and labeled with Vibrant DiD (see Figure 2). Converter cell control groups consisted of CD4^+^CD25^-^ or CD4^+^CD25^+^ cells from control cats incubated in IL2 (100 U/ml) alone for 4 days then labeled with DiD. The positive control for conversion of CD4^+^CD25^-^ Th was a 5d treatment of sorted cells with 5 μg/mL ConA and 10 ng/mL soluble TGFβ which has been described in previous conversion experiments [17]. The target cells for testing conversion cell immunosuppressive function consisted of autologous unlabeled CD4^+^CD25^-^ T cells freshly purified from peripheral blood and stimulated with 5 μg/mL ConA and 100 U/ml IL2 for 4 h. The target cells were washed and added to the converter cell cultures at a converter to target cell ratio of 1:2. Cultures were kept at 37°C for 5 days in RPMI 1640 supplemented with 10% heat-inactivated FBS, 50 μM 2-ME, 1 mM sodium pyruvate, 2 mM L-glutamine, 100 U/ml penicillin, 100 mg/ml streptomycin, and 10U/ml IL2. After 5 days, DiD positive cells were FACS depleted and the remaining cells were used in suppression assays, cell surface molecule analysis, and mRNA analysis. The purity of DiD-depleted cells was >98% as verified by flow cytometric analysis. For conversion blocking experiments, 100 μg/ml anti–TGFβ or 100 μg/ml anti-GARP was added to the converter cell population, or 100 μg/ml anti–TGFβ−RII was added to the target cell population just prior to co-culture. Experimental design is outlined in Figure 2.

### In vitro T cell IL2 suppression assay

Peripheral lymph node (PLN) cells from FIV-negative cats were FACS purified into CD4^+^CD25^-^ target cells and CD4^-^CD8^-^ APCs, combined at a 1:1 ratio, stimulated for 4 h with 5 μg/ml ConA, washed twice in RPMI 1640, and plated at 2 × 10^6^ cells/mL in 12-well plates. DiD negative cells from the conversion assays described above were added as suppressor cells at a suppressor to target cell ratio of 1:2. Effector cell controls for the suppressor assay consisted of CD4^+^CD25^+^ cells from control cats that had been stimulated for 4 days with 10 μg/ml LPS and 100 U/ml rhIL2 (positive control) or non-stimulated CD4^+^CD25^-^ cells from control cats (negative control) as suppressor cells. Controls for IL2 production consisted of ConA stimulated CD4^+^CD25^-^ cells plus APCs without effector cells (positive control) and non-stimulated CD4^+^CD25^-^ cells plus APCs without effector cells (negative control). After 24 hours, 100 uL of the supernatant from each well was analyzed in triplicate by IL2 ELISA using the Feline IL2 DuoSet (DY1890 R&D Systems, Minneapolis, MN) as per manufacturer’s protocol.

### In vitro T cell proliferation suppression assay

Enriched CD4^+^CD25^-^ target cells (10^6^ cells/ml) were stimulated for 4 h with 5 μg/ml Con A, washed twice in RPMI 1640, and plated at 5 × 10^4^ viable cells/well in 96-well U bottom plates. DiD negative cells from the conversion assays described above were added as suppressor cells at suppressor to target cell ratios ranging from 0.125:1 to 1:1. Controls for the suppressor assay consisted of CD4^+^CD25^+^ cells from control cats that had been stimulated for 4 days with 10 μg/ml LPS and 100 U/ml rhIL2 (positive control) or non-stimulated CD4^+^CD25^-^ cells from control cats (negative control) as suppressor cells. Effector and target cells were co-cultured at 37°C for 72 hrs, pulsed with 1 μCi of [^3^H]TdR/well for the last 18 h and harvested using a Filtermate Harvester (Packard Bioscience, Meriden, CT). [^3^H]thymidine incorporation was measured using a Top Count NXT Microplate scintillation counter (Packard Bioscience). Percent inhibition of proliferation was determined based on proliferation of CD4^+^CD25^-^ target cells alone and calculated as follows:

$$\begin{array}{l} \mathrm{percent}\;\mathrm{inhibition}=[\left(\mathrm{CD}4^{+}\mathrm{CD}25^{-}\mathrm{alone}-\mathrm{CD}4^{+}\mathrm{CD}25^{+}\right)\mathrm{cpm}\\ \;/\left(\mathrm{CD}4^{+}\mathrm{CD}25^{-}\mathrm{alone}\right)\;\mathrm{cpm}]\;x100 \end{array}$$

For suppressor activity blocking experiments, the suppressor cells were pretreated with 100 μg/ml anti-TGFβ for 30 minutes then washed, counted, and added to the target cells. Assays were run in triplicate.

### Reverse transcription PCR analysis of FoxP3 and GARP

FoxP3 mRNA was detected by RT-PCR using feline specific primers for FoxP3 or GARP as described previously (Miller et al., 2013, Petty et al., 2008). GAPDH mRNA expression was used as a normalizing control. Briefly, total RNA was isolated from 1 × 10^6^ CD4^+^CD25^+^ or CD4^+^CD25^-^ T cells using RNeasy Protect Mini Kit (Qiagen, Valencia, CA). Reverse transcription was carried out using a reverse transcription system kit from Promega as per the manufacturer’s protocol, followed by PCR using HotStar Taq polymerase (Qiagen, Valencia CA). PCR products were resolved on agarose/ethidium bromide gels and visualized using GelRed (Biotium, Hayward CA).

### Statistical analysis

The Mann–Whitney U test (t test for nonparametric data) was used for pair-wise comparison of parameters. Differences were considered to be significant at p < 0.05.

## Abbreviations

FIV: Feline immunodeficiency virus; GARP: Glycoprotein a repetitions predominant; LN: Lymph node; PBMC: Peripheral blood mononuclear cell; mTGFβ: Membrane-associated TGFβ; TGFβRII: TGFβ receptor II; Treg cell: CD4^+^CD25^+^ T regulatory cell.

## Competing interests

The authors declare that they have no competing interests.

## Authors’ contributions

MM performed all experiments and data analysis except for proliferation assays utilizing [^3^H]thymidine and the PCR analysis displayed in Figure 5E which were performed by CP. CP created the first draft of the manuscript while MM authored the final manuscript and designed Figure 2. JF and MT provided assistance with experimental concepts and design and manuscript preparation. All authors read and approved the final manuscript.
